# Microfluidic diffusional sizing in bioanalysis and biosensing

**DOI:** 10.1039/d5lc01038a

**Published:** 2026-08-26

**Authors:** Lena Bauernhofer, Jasmin Baron, Sandro Keller, Georg Krainer

**Affiliations:** a Biophysics, Institute of Molecular Biosciences (IMB), NAWI Graz, University of Graz Graz Austria sandro.keller@uni-graz.at georg.krainer@uni-graz.at; b Field of Excellence BioHealth, University of Graz Graz Austria; c BioTechMed-Graz Graz Austria; d Gottfried Schatz Research Center, Division of Medical Physics and Biophysics, Medical University of Graz Graz Austria

## Abstract

Microfluidic diffusional sizing (MDS) has emerged as a versatile lab-on-a-chip technology for quantitative analysis of biomolecules in solution, particularly for determining particle sizes and characterizing biomolecular interactions. By measuring the diffusive behavior of molecules under laminar flow in a microfluidic chip, MDS enables the precise determination of hydrodynamic radii and binding affinities, even in complex biological samples, while requiring only minute sample volumes. As a result, MDS is establishing itself as a key tool in the life sciences. This review summarizes recent advances and applications of MDS in the context of bioanalysis and biosensing. We outline the fundamental principles of MDS and highlight its wide range of applications, including studies of protein aggregation, antibody affinity profiling, protein–lipid interactions, and nanoparticle sizing. We further discuss recent technological developments, such as single-molecule detection, label-free strategies, and multidimensional analysis, that have significantly expanded the scope, sensitivity, and utility of MDS. We conclude with an outlook on the anticipated impact of MDS on biomedical research, diagnostics, and therapeutic development.

## Abstract 2

Microfluidic diffusional sizing (MDS) enables determination of molecular size (*R*_h_), binding affinity (*K*_D_), and stoichiometry (*n*) across diverse applications from protein interactions and folding to lipid-particle sizing.

## Graphical abstract

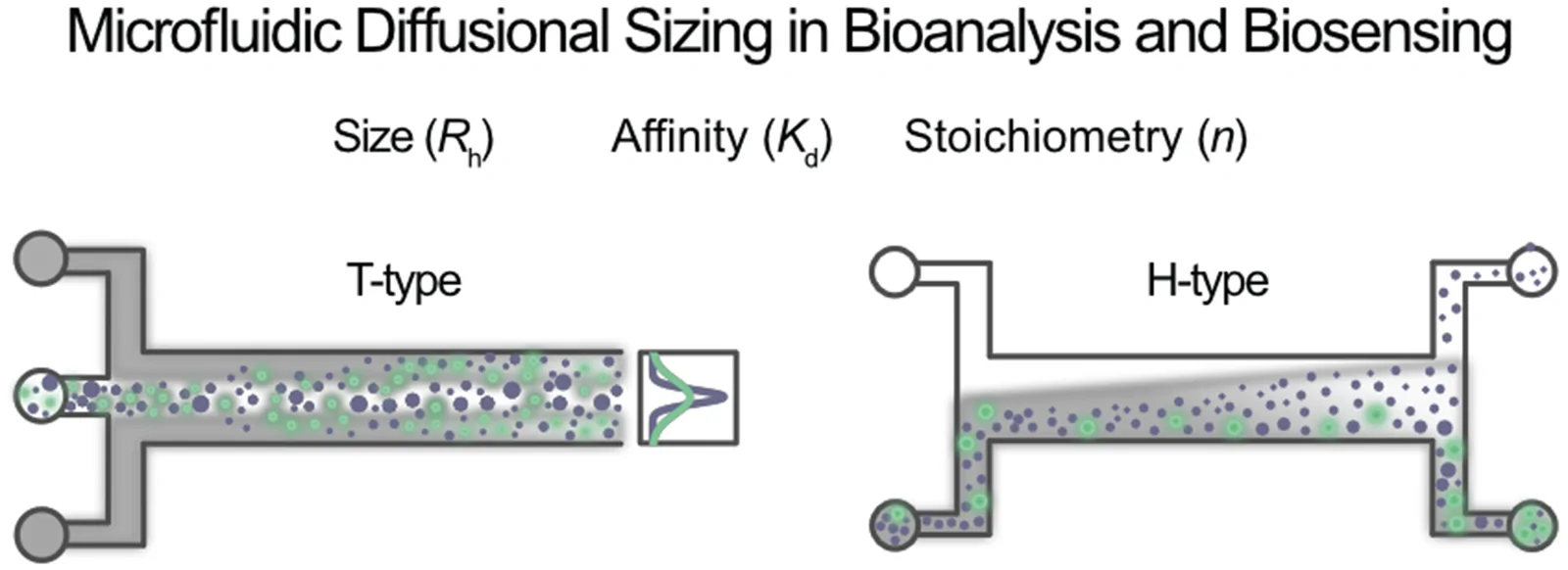

## 1. Introduction

Microfluidic techniques have become essential tools in the life sciences, enabling manipulation of fluids on the micrometer scale.^1–4^ These techniques enable the analysis of ultra-low sample volumes in the sub-microliter range and, when combined with advanced optical technologies, can even detect individual molecules.^5–7^ Microfluidics can be used for the analysis of biomolecules,^8–10^ biomolecular complexes,^11,12^ and cells,^13–17^ and for conducting in-solution studies of non-covalent interactions, which are key to understanding complex biological systems. By integrating preparative and analytical steps on a single chip, microfluidic platforms improve both efficiency and resource utilization while simultaneously enhancing resolution and throughput. Moreover, these platforms are gaining prominence in fields such as synthetic biology,^18,19^ chemical synthesis,^20,21^ and biomedicine.^22,23^

Among microfluidic methods, microfluidic diffusional sizing (MDS)^24^ has emerged as a powerful lab-on-a-chip technique specifically designed for precise measurement of particle sizes, interactions, and association states of fluorescent analytes in solution. By monitoring the diffusion-driven transfer of analytes between phases under laminar flow, MDS provides information about the hydrodynamic radius (*R*_h_)^24^ in microliter samples with nanomolar analyte concentrations, allowing the study of proteins, DNA, RNA, lipid nanoparticles, and other biomolecules or complexes. MDS enables the study of protein and other biomolecule interactions, affinities, and association states, including drug-target binding, as well as the unfolding and aggregation of pathogenic proteins. Moreover, MDS functions reliably even in complex, heterogeneous, and unpurified biological samples, underscoring its role in diagnostic and clinical applications.^25^

Here we review recent advances in MDS in bioanalysis and biosensing. We first give a short introduction to the fundamentals of MDS, including its theoretical background and experimental implementations. We then describe various applications of MDS, including the analysis of protein–protein and protein–nucleic acid interactions, protein folding, misfolding, and aggregation, affinity profiling of proteins such as antibodies, as well as protein–lipid interactions and lipid particle sizing. Next, we highlight recent technical developments that have enabled ultrasensitive detection and high size resolution through digital counting. We conclude by discussing future directions for MDS in bioanalysis and biosensing.

## 2. Fundamentals of MDS

### 2.1. Principle and physical basis of MDS

MDS determines particle size by quantifying the lateral spreading of analytes from a sample stream into one or two adjacent buffer streams during downstream transport in a microfluidic channel.^24^ Due to laminar flow conditions, this spreading is governed primarily by diffusion: smaller particles diffuse further across the channel, whereas larger ones remain closer to their original streamline. The resulting spatial concentration profile provides a readout of the analyte's diffusion coefficient and, consequently, its hydrodynamic size (*i.e.*, *R*_h_).

The physical basis of MDS exploits the distinctive fluid behavior at the microscale under laminar flow conditions, which differ fundamentally from those observed in bulk solutions.^26,27^ At the microscale, viscous forces dominate over inertial forces, so flow remains laminar rather than turbulent. This behavior is described by the Reynolds number (Re),^3,24,28–30^ a dimensionless parameter that compares inertial and viscous forces and is given by Re = *hv*/*ν*, where *v* denotes the velocity, *ν* the kinematic viscosity of the fluid, and *h* the characteristic channel dimension.^29,31^ While laminar flow is generally observed below a critical Reynolds number of approximately 2000, microfluidic experiments, including MDS, typically operate at Re ≪ 1.

Consequently, mixing between adjacent streams occurs almost exclusively by diffusion. Particles therefore spread laterally across the channel while being transported downstream by the flow. The characteristic time (*t*) required for a particle to diffuse over a distance *h* is given by *t* = *h*^2^/*D*, where *D* is the diffusion coefficient.^29^ Faster-diffusing, smaller particles spread more rapidly, whereas larger particles diffuse more slowly and remain closer to their original streamline over the same residence time.

The balance between downstream transport by flow and lateral spreading by diffusion is captured by the Péclet number (Pe = *vh*/*D*), where *v* is again the flow velocity, *h* the characteristic channel dimension, and *D* the diffusion coefficient.^27,29^ MDS experiments are typically operated at high Péclet numbers, meaning that advective transport along the channel dominates over diffusive transport across the channel. As a result, complete mixing is not achieved over short channel distances and may occur only much further downstream, for example near or beyond the outlet.

Importantly, this incomplete, diffusion-limited mixing is the central principle of MDS: the extent of lateral spreading over a defined transport distance reports on the diffusion coefficient of the analyte. By quantitatively analyzing this diffusion process, *D* can be determined and converted into particle size (*i.e.*, *R*_h_), using the Stokes–Einstein relation:
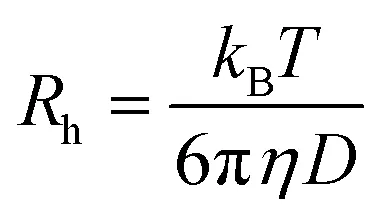
where *k*_B_ is the Boltzmann constant, *T* the absolute temperature, and *η* the dynamic viscosity of the solution.

### 2.2. Evolution and experimental implementation of MDS

MDS evolved from earlier diffusion-based microfluidic co-flow devices that used laminar flow to separate or characterize particles according to their diffusion coefficients.^29,31–34^ One such design is the T-sensor, inspired in part by field-flow fractionation. This early co-flow platform enabled quantification of molecular interactions and analyte concentrations by quantifying the fluorescence intensities within a region of interdiffusion and comparing them with analytical diffusion models predicting diffusion coefficients and kinetics of complex formation.^31,33,34^ A related two-inlet, two-outlet microchannel geometry, termed H-filter, was originally developed for continuous extraction and separation of analytes, establishing the principle that differential diffusivity can serve as a separation and sizing mechanism at the microscale.^29^ These devices laid the conceptual and physical groundwork for what would later evolve into modern MDS, whose channel geometries still closely resemble these early designs. Current MDS implementations retain this basic co-flow architecture but combine it with sensitive optical readout, such as wide-field fluorescence microscopy, to quantify diffusion profiles.

**Fig. 1:**
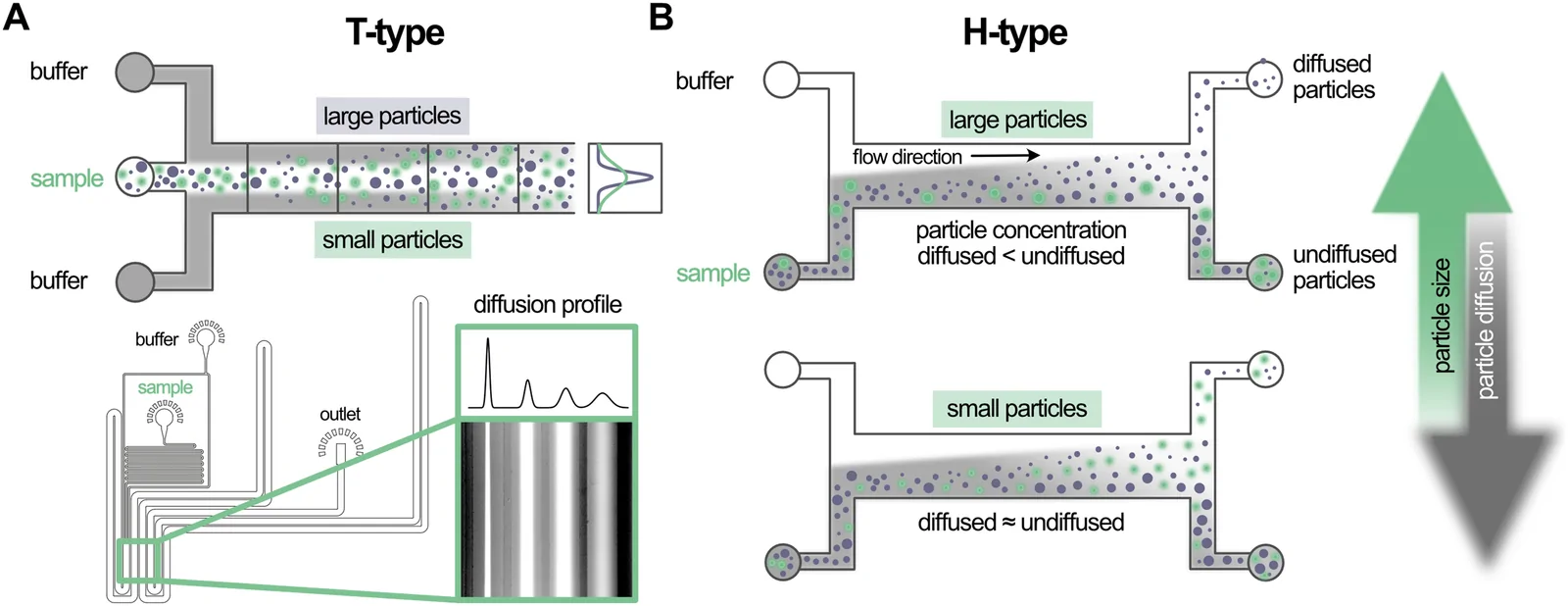
Principle and microfluidic design of MDS. Two types of MDS setups with different channel arrangements are in use. (A) In the three-phase T-type geometry, an analyte stream containing the particles of interest is introduced through the sample inlet and hydrodynamically focused between two auxiliary buffer streams supplied through the buffer inlet. Fluorescence intensity profiles are recorded at multiple positions along the observation channel, corresponding to increasing diffusion times, to monitor the lateral spreading of particles and determine their hydrodynamic radius (*R*_h_). The profiles are acquired by wide-field fluorescence microscopy and analyzed by comparison with numerical simulations of advective–diffusive particle transport. The observation channel terminates at an outlet connected to a syringe pump, which drives the sample and buffer streams through the device by applying negative pressure. (B) In the two-phase H-type geometry, used in commercialized MDS systems, an analyte stream flows alongside a single buffer stream. Sample and buffer solutions are introduced through separate microchannels and brought into side-by-side contact under laminar-flow conditions within a diffusion chamber. After a defined interaction time, the streams are separated again into two outlet channels, and the fluorescence intensity in each channel is measured. *R*_h_ is determined from the ratio of the measured fluorescence intensities. Particle sizes obtained with this geometry represent population-averaged values, as the simplified two-phase arrangement provides lower resolution than the three-phase geometry. In both configurations, the analyte is fluorescently labeled prior to measurement, either covalently, for example using maleimide or click chemistry, or non-covalently, for example through hydrophobic interactions.

Two MDS flow setups with different channel arrangements are currently in use (Fig. 1). Originally, MDS was implemented in a three-phase geometry (T-type; Fig. 1A), where an analyte stream is flanked by two auxiliary buffer streams.^24^ Fluorescence intensity profiles are measured at various points along the microfluidic channel, each corresponding to a different diffusion time that reflects the diffusive spreading of the analytes perpendicular to the flow direction.^24^ These experimental fluorescence profiles are then compared to numerical simulations of the advection–diffusion equation, which describes mass transport under steady-state flow conditions in microfluidic channels.^35–37^

A current, commonly used T-type chip design is shown in Fig. 1A. These devices contain two inlets, one for sample injection and one for the co-flowing buffer solution. Both streams are guided through channels with a height of 25 μm and a width of 50 μm to an entry nozzle, where they merge into a 25 μm high and 225 μm wide observation channel. The nozzle geometry establishes an approximately 1 : 8 sample-to-buffer volume ratio, enabling controlled lateral diffusion of the analyte into the buffer stream. Diffusion is monitored along a folded observation channel of approximately 90 mm in length, which terminates at a waste outlet connected to a syringe pump applying negative pressure. The devices are operated on a wide-field fluorescence microscope, and MDS diffusion profiles are extracted by image analysis.

Subsequent developments in MDS technology and commercialization of MDS instruments led to the adoption of a two-phase geometry (H-type; Fig. 1B), where the analyte stream flows alongside a single buffer stream. The two streams are introduced side by side into a diffusion chamber within the microfluidic channel, where analyte molecules diffuse laterally into the buffer stream. Following a defined diffusion time, the two streams are separated into two outlets, and the fluorescence intensity in each outlet is measured. Determination of diffusion coefficients and hydrodynamic radii is achieved by combining the fluorescence intensity ratio with known flow parameters.^25,28,38^

Commercial H-type instruments, such as the Fluidity One-M (Fluidic Sciences), employ disposable microfluidic chips with channel dimensions in the tens-of-micrometers range and short diffusion path lengths optimized for rapid measurements at moderate flow rates. While specific channel geometries are proprietary, these devices generally integrate sample and buffer inlets, a diffusion chamber in which the two streams come into side-by-side contact, and two outlet channels. An on-chip detection scheme quantifies the fluorescence intensity ratio between the outlet streams, from which diffusion coefficients and hydrodynamic radii are extracted.

As two-phase channel arrangements are limited to average size measurements because of their detection geometry, they are restricted to a size range of *R*_h_ = 1–25 nm, which includes most protein monomers and smaller protein complexes. By contrast, three-phase systems can resolve the full spatial concentration profile within mixed-species samples and extend the size range to particles as large as 250 nm in radius.^24,38^

Beyond these foundational designs, MDS belongs to a broader class of diffusion-governed microfluidic techniques, including Taylor dispersion analysis (TDA),^39^ and flow-induced dispersion analysis (FIDA),^40^ as well as various hybrid platforms that exploit diffusive transport for molecular separation and characterization.^41^ For a broader perspective on microfluidic approaches to studying protein interactions, including both diffusion-based and droplet-based methods, the reader is referred to recent reviews.^3,42^

### 2.3. Probing biomolecular interactions with MDS

Apart from particle size determination, MDS is a quantitative tool for detecting and analyzing molecular binding interactions. The fundamental principle here relies on the fact that binding between two analytes, such as a protein and its ligand, results in the formation of a larger complex, which is reflected in an increase in the size (*R*_h_) of the population average. This increase can be robustly detected by MDS when the interacting molecules differ substantially in size; typically, an *R*_h_ difference of more than 1 nm is sufficient.

Building on this principle, MDS can also be employed to quantify binding affinities, which are typically expressed in terms of dissociation constants (*K*_D_).^3^ This is achieved through titration experiments, in which one binding partner is added stepwise to a constant concentration of the other. As the titrant concentration increases, the average *R*_h_ increases accordingly due to complex formation. Once all binding sites are saturated, *R*_h_ reaches a plateau. The resulting sigmoidal binding curve can be analyzed to extract a binding isotherm, from which *K*_D_ can be accurately determined. For a 1 : 1 binding interaction, the observed hydrodynamic radius at a given titrant concentration [B] is given by:
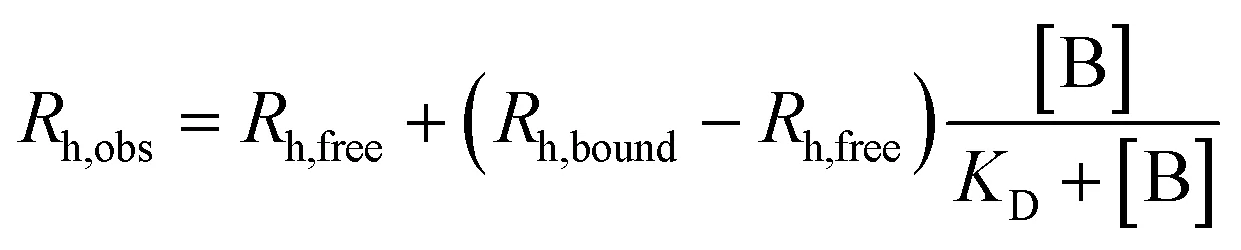
where *R*_h,free_ and *R*_h,bound_ are the hydrodynamic radii of the unbound and fully bound probe, respectively, and [B] is the free titrant concentration. More complex binding models incorporating cooperativity or multiple binding sites require additional parameters and are discussed in section 2.6. Notably, these measurements require labeling of only one binding partner and avoid surface immobilization.

### 2.4. Probing stoichiometry, kinetics and separation with MDS

In addition to measuring binding affinities, MDS also allows determination of complex stoichiometries by analyzing how *R*_h_ changes as a function of analyte concentration.^24,43^ Moreover, it enables determination of unknown analyte concentrations in solution based on their diffusion behavior.^43–47^ In addition to equilibrium measurements, MDS can be implemented in a time-resolved manner to study kinetics, such as the rates of association or dissociation in binding reactions, offering valuable insight into both the thermodynamic and kinetic properties of biomolecular interactions.^48,49^ These features collectively make MDS a versatile tool for analyzing non-covalent interactions across a wide range of biological and bioanalytical contexts.

Furthermore, the differential diffusion behavior underlying MDS inherently enables a separation capability, where species with distinct diffusion coefficients become spatially resolved across the channel width after a defined diffusion time. This principle has been exploited in early diffusion-based microfluidic systems, including the H-filter geometry, for continuous extraction and separation of analytes from complex samples such as whole blood.^29,31^

### 2.5. Labeling in MDS

In conventional fluorescence-based MDS, detection of molecular diffusion generally requires fluorescent labeling of the probe; label-free MDS based on intrinsic fluorescence has also been introduced and is discussed in section 4.1. Typically, labeling is achieved by covalently attaching a fluorophore to the molecule of interest, ensuring that its diffusion can be selectively monitored even in complex mixtures. For proteins, this is most commonly achieved using maleimide- or NHS ester-functionalized dyes, which react with cysteine residues or free amines, respectively, including the N-terminus and lysine side chains. The choice of fluorophore must be carefully considered to avoid altering the molecular properties or interfering with the binding interaction under study. Attaching a fluorescent label to one of the binding partners can significantly alter its physicochemical properties, including its binding affinity and association behavior. Importantly, for protein interaction studies, only one of the interacting partners, which is usually the smaller probe molecule, needs to be labeled, with the binding partner remaining unlabeled. This asymmetric labeling approach minimizes perturbation of the system and allows for highly specific measurements. Additionally, recent advances have enabled non-covalent labeling strategies, either by labeling after separation^38^ or through universal approaches,^25^ such as the use of environment-sensitive dyes that fluoresce only when incorporated into specific molecular environments. These developments have further broadened the range of analytes that can be studied using MDS.

### 2.6. Advantages and limitations of MDS

MDS offers several advantages for measuring particle sizes and studying molecular interactions, particularly in biologically complex media. A key strength is that MDS is performed fully in solution and therefore avoids immobilization of one interaction partner. This distinguishes it from surface-based methods such as enzyme-linked immunosorbent assays (ELISA), surface plasmon resonance (SPR) spectroscopy and biolayer interferometry (BLI). By avoiding surface attachment, MDS minimizes steric hindrance of epitopes and binding sites, reduces surface-induced artifacts, and enables thermodynamic and kinetic experiments under near-native conditions.

Another important advantage is the low sample requirement. MDS typically requires only small sample volumes, around 5–10 μL, and can operate at low analyte concentrations in the nanomolar range. This makes it particularly attractive for precious biological and clinical samples. In addition, because MDS is based on diffusion rather than bulk scattering intensity, it can provide robust hydrodynamic size information in heterogeneous or high-background samples, where methods such as dynamic light scattering (DLS) are often limited by large-particle bias and their requirement for essentially unimodal size distributions.

Despite these advantages, MDS also has several limitations. The method primarily reports hydrodynamic size, typically expressed as the hydrodynamic radius *R*_h_, and does not provide detailed structural information. Conversion of diffusion coefficients into *R*_h_ values *via* the Stokes–Einstein relation assumes spherical, rigid particle behavior in a continuous medium. This represents an approximation for many biomolecules, especially elongated, flexible or intrinsically disordered proteins, for which apparent *R*_h_ values may not directly correspond to true geometric dimensions.^50^ Nevertheless, the apparent *R*_h_ value of a non-spherical or disordered molecule remains a well-defined physical quantity that reports on the ensemble-averaged hydrodynamic drag and can be interpreted quantitatively within polymer physics frameworks. The accessible size range also depends on the microfluidic geometry. H-type MDS systems are generally best suited for particles of approximately 1–25 nm *R*_h_, whereas T-type systems can extend the measurable range up to around 250 nm. Very small molecules, including most drug-like compounds with *R*_h_ values below 1 nm, and very large particles such as extracellular vesicles are therefore difficult or unsuitable for analysis with standard MDS formats. Compared with other microfluidic methods such as FIDA, MDS can therefore be more restricted in size range and size resolution, particularly in H-type geometries and two-phase systems.

A further limitation is the requirement for fluorescence-based detection. In most protein-interaction studies, one binding partner is labeled with a fluorophore, often the smaller probe molecule. Although this allows selective tracking in complex mixtures, fluorescent labeling can alter physicochemical properties, binding affinity or association behavior. Careful control experiments are therefore required to ensure that labeling does not perturb the system under investigation.

Quantitative analysis also relies on several model assumptions. Binding-affinity measurements assume that the system has reached thermodynamic equilibrium during the measurement window (seconds to minutes); very slow association or dissociation kinetics may violate this assumption. Extraction of *K*_D_ values from titration experiments commonly relies on simplified binding models, such as 1 : 1 stoichiometry, whereas cooperative, multivalent or higher-order interactions require more complex modelling and can increase parameter uncertainty. In H-type geometries, the measured *R*_h_ represents a population-weighted average over all species present, making the method sensitive to small amounts of large species such as aggregates, while low-abundance species may be masked by the dominant population. T-type configurations can partly address this limitation by deconvolving the full spatial diffusion profile but may introduce fitting ambiguities when size populations are closely spaced.

Finally, reproducibility across microfluidic chips, instruments, and analysis workflows remains an important area for further standardization, particularly if MDS is to be applied in clinical or diagnostic settings.

### 2.7. Comparison of MDS to other techniques

The strengths and limitations of MDS are best understood in comparison with complementary sizing and interaction-analysis methods (Table 1).

**Table 1:** Comparative overview of MDS and alternative hydrodynamic sizing and interaction analysis techniques. Summary of measurement principles, accessible size ranges, species resolution, sample requirements, labeling and immobilization constraints, compatibility with complex biological media, and practical performance considerations. MDS (H-type and T-type geometries) is highlighted in comparison with established ensemble and single-molecule methods including DLS, FIDA, FCS, NTA, AUC, SEC-MALS, SPR/BLI, and MST. Abbreviations: fluorescence (fl.), intrinsic fluorescence (intr. fl.), UV absorbance (UV abs.), immobilized (imm.)

Feature	MDS (H-type)	MDS (T-type)	FIDA	DLS	FCS	NTA	AUC	SEC-MALS	SPR/BLI	MST
Measurement principle	Lateral diffusion	Lateral diffusion	Taylor dispersion	Intensity autocorrelation	Fluorescence autocorrelation	Particle tracking	Sedimentation	Elution + scattering	Surface binding kinetics	Thermophoresis
Size range (*R*_h_)	∼1–25 nm	∼1–250 nm	∼0.5–1000 nm	∼0.5–1000 nm	∼0.5–100 nm	∼15–500 nm	∼1–100 nm	∼1–50 nm	N/A	N/A
Species resolution	Population average	Multimodal deconvolution	Up to 3 species	Biased to large species	Single species (dilute)	Individual particles	High (c(s) dist.)	Chromatographic	N/A	N/A
Sample volume	∼5–10 μL	∼10–50 μL	∼5 μL	∼10–50 μL	∼10–50 μL	∼300 μL	∼100–400 μL	∼50–100 μL	∼100 μL	∼5–10 μL
Concentration range	nM–μM	nM–μM	nM–μM	μM–mM	pM–nM	∼10^8^–10^9^ particles per mL	μM	μM	nM–μM	nM–μM
Labeling required	Yes (fl.)	Yes (fl.)/no (intr. fl.)	Yes (fl.)/no (intr. fl./UV abs.)	No	Yes (fl.)	No (or fl.)	No	No	No (imm.)	Yes (fl.)
Surface immobilization	No	No	No	No	No	No	No	No	Yes	No
Complex media compatible	Yes	Yes	Yes	Limited	Limited	Limited	No	No	Limited	Yes
*K*_D_ determination	Yes	Yes	Yes	No	Limited	No	Limited	No	Yes	Yes
Measurement time	∼min	∼min	∼min	∼s	∼s–min	∼min	∼hours	∼30–60 min	∼min	∼min
Throughput	Medium (∼60 h^−1^)	Low (<5 h^−1^)	Medium (∼10–20 h^−1^)	Medium–high	Low–medium	Medium	Low	Medium	Medium–high	Medium
Relative instrument cost	Low–mod.	Moderate (custom)	Low–mod.	Low	High	Moderate	High	High	Mod.–high	Moderate

Among hydrodynamic sizing techniques, dynamic light scattering (DLS) provides rapid and label-free measurements but is strongly biased toward larger species due to intensity-weighted scattering and therefore performs poorly in polydisperse or heterogeneous samples. MDS, by contrast, provides diffusion-based sizing of a fluorescently detected species and can operate more robustly in complex, turbid, and heterogeneous biological media.

Flow-induced dispersion analysis (FIDA), which measures Taylor dispersion in thin capillaries, offers a broader size range (∼0.5–1000 nm) and higher size resolution than H-type MDS. It can also resolve multiple coexisting species under favorable conditions.^28,40^ While FIDA typically requires longer acquisition times (minutes per sample) and more complex fitting procedures, it can operate in a label-free mode using intrinsic UV absorbance detection and offers higher size resolution than MDS, making the two methods largely complementary.

Fluorescence correlation spectroscopy (FCS) can determine diffusion coefficients at the single-molecule level with excellent sensitivity. Compared with MDS, FCS offers higher temporal resolution and sensitivity, but is generally limited to highly dilute and optically controlled environments requiring specialized confocal optics and careful analysis.^51^

Nanoparticle tracking analysis (NTA) enables direct visualization and sizing of individual particles but is restricted to particles larger than approximately 30 nm and provides limited information on molecular interactions.^52^ MDS, in contrast, is better suited for smaller biomolecules and protein complexes in the low-nanometer *R*_h_ range, but does not directly visualize individual particles.

Analytical ultracentrifugation (AUC) and size-exclusion chromatography coupled to multi-angle light scattering (SEC-MALS) are gold-standard methods for detailed size characterization, oligomerization analysis, and mass determination. Compared with MDS, they provide richer biophysical information and, in the case of SEC-MALS, absolute molar mass estimates. However, they require relatively large sample volumes, longer measurement times, and more purified samples. SEC-based methods may perturb weak interactions through dilution and column separation, whereas MDS can probe interactions directly in solution and at lower sample consumption.^53,54^

For binding affinity measurements, surface plasmon resonance (SPR) and biolayer interferometry (BLI) are widely used but require immobilization of one binding partner on a sensor surface, which can introduce steric constraints, mass transport limitations, and avidity artifacts.^55^ Microscale thermophoresis (MST) is performed in solution and can detect binding without immobilization, but the thermophoretic signal depends on multiple physicochemical parameters (size, charge, hydration shell), making it less directly interpretable than the size-based readout of MDS.^56^

## 3. Applications for bioanalysis and sensing

Based on its ability to accurately determine particle sizes and binding affinities in solution, MDS offers a versatile platform for bioanalytical and biosensing applications. The following sections will present an overview of important areas where MDS has been successfully applied, particularly in characterizing protein–protein and protein–nucleic acid interactions, studying protein folding, misfolding, and aggregation, profiling antibody–antigen interactions, analyzing protein–lipid interactions, and sizing lipid particles.

### 3.1. Protein–protein and protein–nucleic acid interactions

**Fig. 2:**
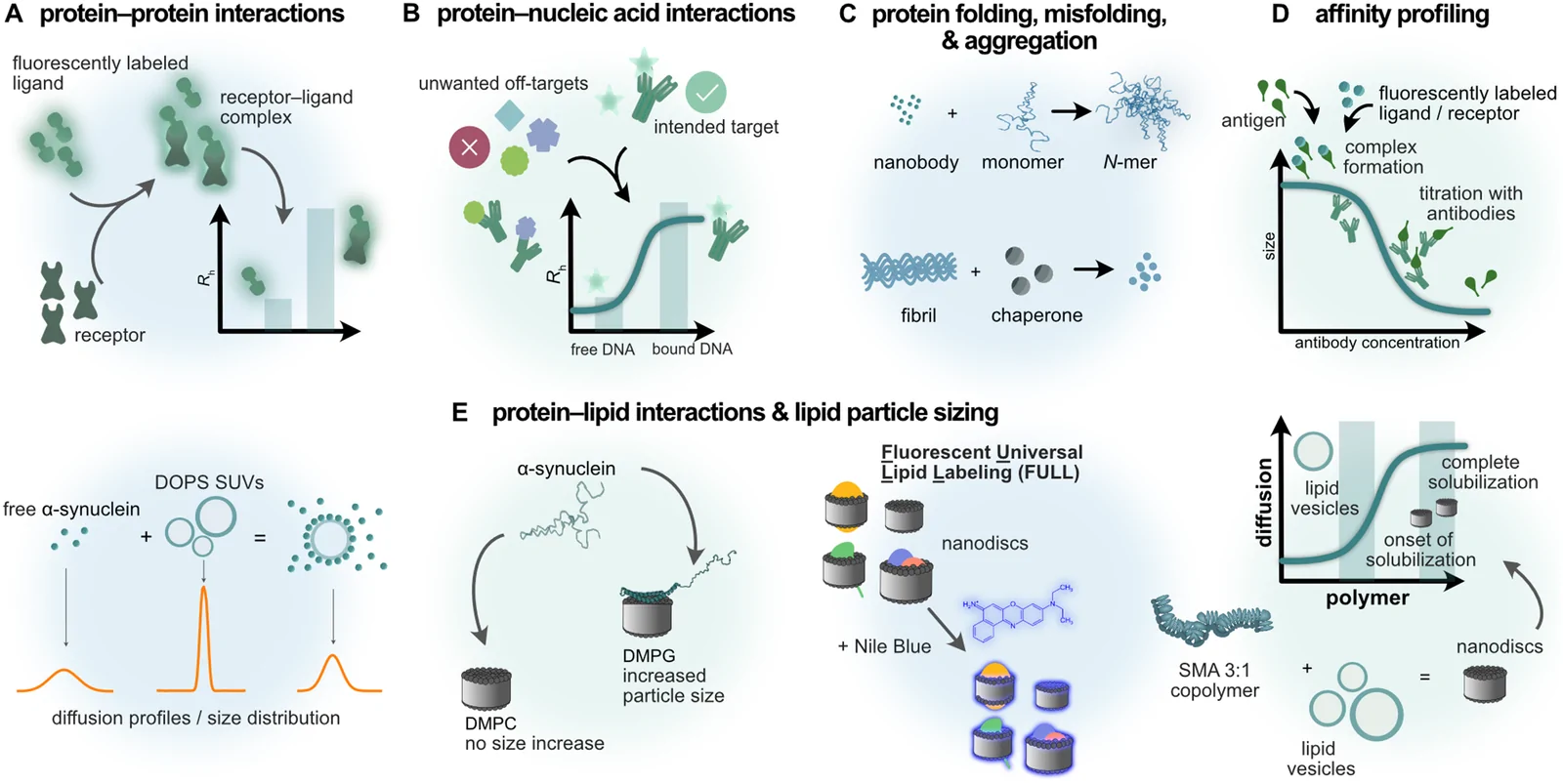
Applications of MDS. (A) Protein–protein interactions (PPIs). MDS enables detection of PPIs, such as receptor–ligand complex formation, by measuring the changes in hydrodynamic radius (*R*_h_) upon binding of a fluorescently labeled ligand to its receptor. Typically, the smaller binding partner is fluorescently labeled, as the resulting size increase is more pronounced. (B) Protein–nucleic acid interactions. MDS is also well suited to detect interactions between proteins and nucleic acids, such as RNA and DNA.^74,76^ For instance, nonspecific interactions of antibodies and off-target molecules (*e.g.*, ssDNA) can be detected.^76^ Adapted from ref. 76 with permission from the *Proceedings of the National Academy of Sciences USA*. Copyright © 2023 the authors. (C) Protein folding, misfolding, and aggregation. MDS has become a useful tool for studying the oligomerization, aggregation, and disaggregation processes of proteins. The technique allows real-time monitoring of these processes and can reveal bimodal size distributions that reflect distinct stages of structural transitions. (D) Affinity profiling. With MDS, binding affinities of two distinct molecular interactions and protein concentrations can be determined directly in complex samples (*e.g.*, serum or blood). Moreover, these assays can be used to monitor competitive binding events by changes in binding affinities and hydrodynamic sizes. Adapted from ref. 43 with permission from the American Chemical Society. Copyright © 2021, the authors. (E) Protein–lipid interactions and lipid particle sizing. MDS can be used to monitor changes in particle size distribution upon protein binding to lipid vesicles or nanodiscs,^116,118^ solubilization of lipid vesicles into polymer-nanodiscs,^130^ or nanoparticle sizing.^25^ Adapted from ref. 116 with permission from the American Chemical Society (ACS). Copyright © 2018, ACS. Adapted from ref. 118 under a CC BY 4.0 License. Copyright © 2022, the authors. Adapted from ref. 25 under a CC BY 4.0 License. Copyright © 2022, the authors. Adapted from ref. 130 with permission from Elsevier B.V. Copyright © 2020, Elsevier B.V.

Protein–protein interactions (PPIs) are fundamental to virtually all cellular processes,^57^ as most proteins carry out their biological functions through interactions with other proteins.^58–60^ Characterizing PPIs and complex formation is therefore essential for understanding both normal functional behavior and aberrant interactions that can lead to dysfunction and disease. MDS has proven to be a robust method for measuring PPIs in solution, detecting binding events through changes in *R*_h_ (Fig. 2A). This enables efficient detection and quantification of PPIs, including their binding affinity.

The most basic PPIs are binary interactions such as the binding of an antibody to its antigen, which has been extensively studied using MDS.^24,38^ Detecting PPIs by MDS is particularly straightforward when the binding partners exhibit a substantial difference in size. For example, MDS was used to study the binding between the protein α-synuclein, which has been implicated in Parkinson's disease, and the nanobody NBSyn87, a single-domain antibody fragment.^24^ Binding events were detected by monitoring the increase in the nanobody's hydrodynamic size upon complex formation. MDS revealed that the nanobody binds to both monomeric α-synuclein and fibrillar forms, as evidenced by a bimodal size distribution corresponding to free nanobody and nanobody–fibril complexes. Notably, the experiments were performed in crude cell lysate containing α-synuclein alongside a heterogeneous mixture of cellular proteins, demonstrating the robustness of MDS in detecting PPIs under native-like conditions.

Since then, MDS has been used to measure a wide range of PPIs. For example, one study used MDS to provide new insights into the complex mechanisms of cytokine–receptor interactions.^61^ Specifically, MDS was used to determine binding affinities (*K*_D_ values) between interleukin-17 (IL-17) isoforms AA, AF, and FF, and their receptors IL-17RA and IL-17RC. The study also investigated how the macrocyclic compound 369, a small-molecule inhibitor, affects receptor affinities by modulating receptor dynamics. Unlike previous optical and spectroscopic approaches^62–71^ relying on immobilized proteins, MDS enabled correlation of receptor affinities with protein flexibility within the IL-17 family. MDS revealed that the small-molecule inhibitor rigidifies IL proteins, especially the isoform IL-17 AA, significantly reducing the affinity of the interleukin-17 isoform for its receptor.

Similarly, MDS has been applied to study the self-assembly of proteins into oligomeric structures in real time. To this end, the self-assembly of the substrate-binding subdomain (SBD641) of human heat shock protein 70 (Hsp70) under native solution conditions was monitored.^48^ MDS enabled direct monitoring of changes in oligomer populations and allowed the dissection of the thermodynamic and kinetic parameters governing the assembly process. The authors demonstrated that the self-assembly of SBD641 is highly cooperative, with strong structural constraints on the size of the resulting oligomers. These constraints are likely imposed by specific molecular interactions at the oligomerization interface, which dictate the geometry and stability of the assemblies.^72^

MDS is also well suited for characterizing PPIs involving membrane proteins. For example, it has been used to identify interactions between human aquaporin (AQP) isoforms and the regulatory protein calmodulin (CaM).^73^ Binding of CaM to AQP0 tetramers embedded in detergent micelles reduced the diffusion coefficient, which reflected an increase in *R*_h_ and allowed the extent of complex formation to be studied within a membrane-mimetic environment. Conversely, when the same experiments were conducted with another aquaporin isoform, AQP2, no changes in diffusivity and, hence, hydrodynamic size were observed, indicating the absence of CaM binding.

In addition to protein–protein interactions, MDS is well suited for studying protein–nucleic acid interactions. For instance, MDS has been employed to investigate the binary interactions between antimicrobial peptides (AMPs) and RNA targets, including 16S and 23S ribosomal RNA.^74^ The experiments revealed that different AMPs (*i.e.*, P113, Os-C, and Buforin-2) are capable of compacting and condensing structured RNAs. Intriguingly, compaction was significant and more pronounced than previously found for other molecules such as spermidine.^75^ This indicates that AMPs engage in specific binding interactions with RNAs, rather than simple charge neutralization of the nucleic acid molecules. Moreover, MDS was used to monitor RNA-induced phase separation, uncovering previously unknown antibacterial modes of action of these AMPs.

In another study,^76^ nonspecific interactions between a library of humanized anti-trinitrophenyl antibodies (HzATNP) and off-target molecules such as single-stranded DNA (ssDNA) were quantified and correlated with protein surface properties (Fig. 2B). The antibody library,^77^ consisting of various isoforms of HzATNP, was chosen to represent a broad range of protein solubilities based on physicochemical surface-property hot spots.^77^ Mutations in these hot spots modulate surface properties such as hydrophobicity, charge, and patch size and, consequently, influence the binding affinities and behavior of these antibodies. By varying the antibody concentration, the authors generated binding isotherms and determined nonspecific binding affinities of antibody–DNA interactions. These experiments showed changes in *K*_D_ spanning multiple orders of magnitude due to different hot-spot mutations.

Together, the above examples demonstrate that MDS is a valuable tool for resolving interactions between proteins, peptides, and nucleic acids. It offers a quantitative approach for studying the thermodynamics and kinetics of interactions and oligomerization in native solutions. However, it should be noted that MDS-derived *K*_D_ values are associated with inherent uncertainties due to limited size resolution and simplified binding models. Orthogonal validation using methods such as isothermal titration calorimetry (ITC), SPR or FCS is recommended for complex systems or subtle binding-induced size changes.

### 3.2. Protein folding, misfolding, and aggregation

An area where MDS has provided deep insights is in protein folding, misfolding, and aggregation. These processes not only underpin protein structure formation^78,79^ but are also implicated in the onset and progression of various neurodegenerative diseases through the conversion of soluble proteins into aggregates (Fig. 2C).^80–82^

For example, in one study,^83^ MDS was used to probe the pH-dependent unfolding of unlabeled bovine serum albumin (BSA). Specifically, the folding and unfolding of BSA was monitored across pH values ranging from 1.2 to 10.2, revealing that BSA remained folded between pH 4.3 and 10.2. Below pH 4.3, unfolding was indicated by a progressive increase in hydrodynamic size. These experiments were consistent with data obtained from circular dichroism (CD) spectroscopy^83^ and previous studies,^84^ further highlighting MDS as a suitable method for investigating protein stability and unfolding. Such experiments are especially interesting for the formulation of protein therapeutics such as clinical monoclonal antibodies (mAbs), where protein stability is a key parameter.

MDS has also been applied in physiological settings relevant to protein folding,^85^ for example, to study the chaperone activity of trigger factor (TF) during co-translational folding. The authors measured TF binding to megadalton ribosome–nascent chain complexes to dissect the thermodynamics underlying TF's role as the sole ribosome-associated chaperone in *E. coli*. The study showed that TF binding to empty 70S ribosomes is enthalpy-driven, with micromolar affinity, whereas binding to nascent chains—whether intrinsically disordered or folding-competent—occurs with nanomolar affinity due to a favorable entropic contribution. These findings suggest a general mechanism for co-translational TF function that relies on occupation of the TF–substrate binding groove, rather than specific sequence complementarity between the chaperone and the nascent chain.

Molecular chaperones assist in protein folding and regulate protein assembly, playing a crucial role in preventing pathological aggregation.^48,86–92^ MDS has helped elucidate the structures and functions of biomolecules associated with such pathologies. For example, the formation of amyloid fibrils of Aβ(M1–42) has been shown to produce toxic oligomers, which are thought to be involved in the progression of Alzheimer's disease (AD).^80–82^ In a recent study,^80^ MDS was used to measure the interaction between the molecular chaperone clusterin and Aβ(M1–42) fibrils. Clusterin exhibits an inhibitory effect on aggregation; therefore, the specific binding interactions between clusterin and fibrils are key to the inhibition of fibril elongation.^80^ Size distributions extracted from MDS diffusion profiles were analyzed in the absence and presence of Aβ(M1–42). While clusterin alone displayed a unimodal size distribution (*R*_h_ ∼ 8 nm), the presence of fibrils resulted in a bimodal distribution, indicating free clusterin and clusterin–amyloid complexes (*R*_h_ > 100 nm).^80^ By contrast, no binding was observed in experiments employing BSA as a non-chaperone control protein. MDS further showed that inhibition originates from the strong binding association of clusterin to fibril ends. However, the binding affinity obtained from MDS (670 nM) was nearly three orders of magnitude weaker than the value estimated from the kinetic analysis (1 nM).^80,93,94^ This discrepancy was attributed to the fact that kinetic analyses are primarily sensitive to interactions with fibril ends, whereas MDS captures the overall affinity, including binding to fibril surfaces. Transmission electron microscopy (TEM) confirmed that clusterin binds to both fibril ends and surfaces.^80^

MDS can also be used to observe and quantify disaggregation processes. For example, the ATP-dependent disaggregation of α-synuclein fibrils by the Hsp70 molecular chaperone machinery, comprising Hsc70, DnaJB1, and Apg2, was monitored under native-like conditions.^49^ Disaggregation was reflected in a broadening of the diffusion profile, indicating a decrease in *R*_h_ due to an increase in the concentration of free monomer. The authors also demonstrated that Hsc70, together with its co-chaperones, can fully reverse α-synuclein aggregation to its soluble monomeric state. The reaction followed first-order kinetics, with monomers being directly removed from the fibril ends, with insignificant contribution from fibril fragmentation. In addition, this study dissected the thermodynamics of the Hsc70-mediated disaggregation process.

Collectively, these studies demonstrate that MDS provides kinetic and thermodynamic information on aggregation and disaggregation processes that is complementary to conventional methods such as thioflavin T (ThT) fluorescence, TEM and CD spectroscopy. However, MDS does not provide direct information on secondary structure or aggregate morphology. Quantification of disaggregation kinetics from diffusion profiles requires further consideration of the heterogeneous size distributions of fibrillar species and potential dependence on the chosen deconvolution approach.

### 3.3. Affinity profiling

An important application of MDS is the affinity profiling of clinical antibodies. Unlike most currently used diagnostic tools that rely on the immobilization of antibodies, MDS can determine antibody affinities, binding stoichiometries, and concentrations directly in solution (Fig. 2D).

MDS has been used to quantify the interactions of murine analogs of therapeutic antibodies (^ch^aducanumab, ^ch^gantenerumab, 3D6 (bapineuzumab), and m266 (solanezumab)) with Aβ42 monomers and fibrils.^95^ The results revealed clear differences in binding preferences and affinities: ^ch^aducanumab exhibited weak monomer affinity (*K*_D_ ∼9 μM) but high affinity (*K*_D_ ∼1 nM) for fibrils, indicating strong fibril specificity. By contrast, m266 exhibited high affinity for monomers (*K*_D_ ∼4 nM) but no measurable binding to fibrils, consistent with its inhibition of primary nucleation. ^ch^gantenerumab and 3D6 both bound to fibrils with higher affinity than to monomers (*K*_D_ ∼30 nM *vs.* 500 nM and 0.3 nM *vs.* 40 nM, respectively), but with distinct stoichiometries reflecting different binding modes—fibril ends for ^ch^gantenerumab and along the fibril axis for 3D6. The measured stoichiometries were one ^ch^aducanumab molecule per ∼4.5 Aβ42 monomers in fibrils, one ^ch^gantenerumab molecule per ∼44 Aβ42 monomers, and one 3D6 molecule per ∼40 Aβ42 monomers, highlighting differences in epitope accessibility and binding orientation. These differences in stoichiometry correlate with the antibodies' abilities to interfere with distinct microscopic steps of Aβ aggregation, offering valuable mechanistic insights into the distinct inhibitory actions of each antibody.

The affinity profiling approach has also been demonstrated in complex media. In a recent study,^45^ alloantibody binding to human leukocyte antigens (HLA), an extensively used clinical biomarker in organ transplantation, was monitored in human serum.^96,97^ The authors showed that MDS enables reliable quantification of both the affinity and concentration of HLA-specific alloantibodies. Using transplant patient sera, they demonstrated that the assay can differentiate alloantibody reactivity even against HLA proteins of highly similar structure, providing information not attainable through other techniques currently used to study clinical samples, such as ELISA^98–101^ and bead-based multiplex assays. Surface immobilization of proteins, which is essential for such methods, often leads to nonspecific interactions and can limit accessibility to soluble binding partners.^102,103^ Consequently, measurements of patient samples are at best semi-quantitative as binding affinities and concentrations are difficult to quantify.^104–106^ These results outline a path toward the detection and in-depth profiling of humoral immunity and may enable new insights into the clinical relevance of antibody reactivity in transplantation and beyond. Importantly, this study also demonstrates that MDS is not impeded by autofluorescence arising from endogenous serum components, such as heme complexes, thereby ensuring accurate signal detection.^107,108^ The study also confirmed that the choice of fluorescent label does not significantly impact affinity measurements, reinforcing the robustness of MDS for clinical immunoprofiling.

Finally, a recent study^109^ evaluated the capacity of natural products to modulate the interaction between SARS-CoV-2 spike RBD and ACE2 proteins, a crucial step in the process of virion entry into host cells.^110,111^ To this end, several compound classes, including flavonoids, anthraquinones, saponins, ivermectin, chloroquine, and erythromycin, were screened as potential inhibitors. Using MDS, the authors monitored changes in the hydrodynamic size of the protein complex (*R*_h_ ∼4.35 nm for the RBD–ACE2 complex) in the presence of the tested compounds. Rather than focusing on the *K*_D_ values, which would require more experimental effort and sample, monitoring changes in complex size proved to be more straightforward for screening purposes. Here, an increase in *R*_h_ indicated a distension or aggregation of the complex, whereas a decrease was interpreted as collapse or partial separation of the RBD–ACE2 complex. MDS screening revealed that a saponin-rich quinoa husk extract strongly increased *R*_h_ (+150%), while naringenin and ivermectin induced smaller increases. In contrast, *Rhei radix* extract and quercetin reduced *R*_h_ (∼−14% and −11%), suggesting disruption of RBD–ACE2 binding. Other tested compounds showed no significant effect, highlighting MDS as a rapid, low-volume method for identifying SARS-CoV-2 entry modulators.

A specific assay for affinity profiling, namely microfluidic antibody affinity profiling (MAAP), enables the independent determination of binding affinities of two distinct molecular interactions. Note that MAAP is not a separate instrument but rather a specific data-analysis framework applied to standard T-type MDS hardware, in which the full spatial diffusion profile is globally fitted to extract both *K*_D_ and the absolute concentration of the binding partner simultaneously. Recently, MAAP was employed to measure the affinities of molecular interactions involved in the neutralizing mechanisms of SARS-CoV-2 cell entry.^43^ Here, binding affinities of the viral spike protein and neutralizing antibodies (NAbs) to angiotensin-converting enzyme 2 (ACE2) were determined. Using MDS, quantitative information on the inhibition of spike protein binding to the ACE2 receptor by patient antibodies was obtained through the measurement of *R*_h_ and *K*_D_. MAAP played an important role in unraveling the diverse immune responses to COVID-19 and in characterizing antibody-mediated inhibition of ACE2–S1 binding, as it furnishes both binding affinities and concentrations of anti-S1 antibodies.

Similarly, in a follow-up study, MDS was used to robustly determine changes in binding affinities of the receptor-binding domains to ACE2 and NAbs due to mutations in the SARS-CoV-2 variants.^44^ Comparing the measured binding affinities and binding events provided structural insights into the variants and how these structural differences, based on mutations, influence interactions of the SARS-CoV-2 variants with NAbs and ACE2. To this end, the authors measured binding affinities of the receptor binding domains (RBD) from the wild type (WT) and the mutated variants RBD-alpha and RBD-beta (N501Y, K417N, and E484K) to ACE2 and monoclonal NAbs. Intriguingly, no binding of RBD-beta to NAbs could be detected, suggesting that the mutation had caused an essential epitope change in the mutant variants.

Additionally, MAAP was used to determine affinities and concentrations of anti-RBD antibodies in sera obtained from individuals who had recovered from an infection with the original SARS-CoV-2 variant.^44^ Compared to standard procedures that measure only antibody titers, MAAP allows the simultaneous determination of both antibody affinities and concentrations.^44,46^ This makes it possible to detect shifts in binding affinity across the entire antibody population and to identify subsets of antibodies with diminished or lost binding capacity. Importantly, the information gained by MAAP provides insights into viral strategies to evade the host's immune response.

In another example, MAAP was used to study and detect cross-reactivity of antibodies against SARS-CoV-2 and common human coronaviruses (HCoVs) in sera.^47^ Here, unlabeled SARS-CoV-2 RBD was added to HCoV-NL63 RBD and antibodies to observe competitive binding. Competitive binding was indicated by a decrease in the hydrodynamic size of the immune complex, as cross-reactive antibodies would likely bind to the excess of unlabeled SARS-CoV-2 RBD. Moreover, MDS could distinguish between antibodies from acute infections *versus* immunological memory, as the binding affinities and concentrations of serum antibodies could be correlated to the humoral immune response. In conclusion, MAAP can be used to closely monitor the induction of immune responses after interventions such as vaccination, and the information gained from such experiments can be used to evaluate antibody production efficacy.

The MAAP approach exemplifies a key advantage of MDS over surface-based methods such as ELISA and bead-based multiplex assays for antibody profiling in clinical samples. By operating in free solution, it enables simultaneous determination of *K*_D_ and antibody concentration from a single titration experiment. However, compared to ELISA, MAAP typically offers lower throughput and requires more advanced data analysis. In addition, the need for fluorescent antigen labeling may limit its applicability when suitable labeling strategies are not available or may perturb the epitope of interest.

### 3.4. Protein–lipid interactions and lipid-particle sizing

Protein–lipid interactions play a crucial role in regulating many cellular functions.^112,113^ Understanding these interactions is essential for gaining mechanistic insights into protein behavior and the functional properties of cellular membranes. The large surface-to-volume ratio of living cells provides a vast landscape for molecular interactions that can modulate protein functions such as binding affinities and self-assembly propensities.^114,115^ MDS has proven to be a useful technique for studying protein interactions with various lipid-bilayer systems (Fig. 2E).

For instance, MDS has been widely applied to investigate protein–lipid interactions using membrane-mimetic vesicles. In a recent study,^116^ MDS was used to study interactions between α-synuclein and negatively charged lipids (DOPS) using small unilamellar vesicles (SUVs), particularly focusing on the thermodynamics of protein–lipid interactions (Fig. 2E). To this end, the relative concentrations of free and membrane-bound α-synuclein were quantified, and the results were interpreted in terms of dissociation constants and binding stoichiometries, defined as the average number of lipid molecules per bound α-synuclein molecule. Similarly, another study^117^ probed the interactions of both monomeric and oligomeric forms of α-synuclein with SUVs and large unilamellar vesicles (LUVs), and showed that oligomeric α-synuclein binds to negatively charged lipid membranes (DOPS) with up to 150-fold higher affinity than the monomeric form. Moreover, the authors demonstrated that oligomeric α-synuclein competes with and displaces monomeric α-synuclein from the membrane surface, potentially disrupting its functional membrane binding.

The application of MDS for analyzing protein–lipid interactions has recently been extended by combining it with polymer-encapsulated lipid-bilayer nanodiscs (Fig. 2E).^118^ This approach enables the quantification of weak, non-stoichiometric binding events between soluble proteins and membrane lipids in a native-like environment. A particularly illustrative application involves the use of electroneutral nanodiscs—formed from sulfobetaine-functionalized copolymers, sulfo-DIBMA and sulfo-SMA—to probe the interactions of α-synuclein and adrenocorticotropic hormone (ACTH) with different phospholipid headgroups. In MDS experiments, both α-synuclein and ACTH specifically bound to nanodiscs containing anionic lipids (*e.g.*, DMPG) but not to those containing zwitterionic lipids (*e.g.*, DMPC), consistent with physiological expectations. These lipid-specific interactions were not detectable using conventional, polyanionic polymers such as diisobutylene-*alt*-maleic acid (DIBMA)^119,120^ or styrene-*co*-maleic acid (SMA)^121,122^ copolymers, which induced nonspecific binding due to unspecific electrostatic interactions. The combination of MDS and electroneutral nanodiscs thus enables precise and interference-free quantification of protein–lipid interactions, opening new avenues for mechanistic studies of membrane-associated processes.

In another study,^123^ MDS was applied to explore the role of a putative phosphatidylinositol-4,5-bisphosphate (PIP_2_) interaction site in the regulation of transient receptor potential ankyrin 1 (TRPA1) channels. Peptide binding was assessed for two segments (L992–N1008 and T1003–P1034) using nanodiscs containing PIP_2_ in a matrix of zwitterionic phospholipids. The data showed that PIP_2_ is essential for TRPA1 lipid binding, as no interaction was observed with pure zwitterionic membranes. Additionally, MDS revealed that calcium ions reduce the binding affinities of both peptides to lipid membranes. This study demonstrated that combining MDS with novel polymer-based nanodiscs provides an effective assay for probing the charge dependence of protein–membrane interactions.

MDS has also proven valuable for the analysis of lipid nanoparticles themselves. One approach is fluorescent universal lipid labeling for MDS (FULL-MDS),^25^ which enables nanoparticle sizing without covalent labeling (Fig. 2E). FULL-MDS relies on the spontaneous incorporation of water-soluble fluorophores such as Nile Red and Nile Blue into lipid nanoparticles. These dyes exhibit low quantum yields in aqueous solution but become highly fluorescent in lipid environments.^124,125^ Therefore, FULL-MDS enables selective size measurement of lipid nanoparticles without covalent fluorophore coupling. The method was established using lipid-bilayer nanodiscs encapsulated by the polymer glyco-DIBMA^126,127^ or the small-molecule amphiphile dodecyl diglucoside (DDDG).^128,129^ The authors further demonstrated that FULL-MDS can size lipid nanodiscs extracted from complex, heterogeneous cell extracts of both prokaryotic and eukaryotic origin.^25^ Overall, FULL-MDS provides an alternative to conventional techniques such as DLS, particularly for accurate sizing in biologically complex and heterogeneous samples.

In addition, MDS has been used to directly monitor nanodisc formation during the solubilization of lipid bilayers using SMA (Fig. 2E).^130^ The authors showed that MDS enables measurement of nanodisc size and determination of the polymer/lipid ratio required to induce vesicle disruption. The same study used MDS to investigate peptide interactions with SMA-encapsulated nanodiscs. Specifically, the authors investigated the aggregating Tau peptide K18 with lipid-bilayer nanodiscs with or without PIP_2_. While nanodiscs lacking PIP_2_ did not promote aggregation, PIP_2_-containing nanodiscs showed strong interaction with K18 and induced aggregation, highlighting MDS as a tool for probing protein aggregation phenomena at lipid membranes.

MDS has also been applied to validate protein-conjugation strategies in polymer nanodiscs. In a recent study,^126^ the glycosylated polymer glyco-DIBMA was used to encapsulate KvAP, a voltage-gated potassium ion channel, in lipid-bilayer nanodiscs under native-like conditions. To assess successful conjugation with the fluorescent dye ATTO-488 maleimide, the diffusion behavior of labeled KvAP was compared to that of the free dye. MDS revealed a marked decrease in the diffusion coefficient upon conjugation, corresponding to an *R*_h_ value of ∼5 nm, confirming covalent attachment of the dye to nanodisc-embedded KvAP. These results show that glyco-DIBMA nanodiscs are compatible with thiol-reactive labeling and MDS-based analysis of membrane proteins in a near-native environment.

Another recent and noteworthy development in the analysis of lipid nanoparticles is FluoMDS,^131^ a fluorescence-based MDS approach that integrates multi-wavelength fluorescence detection to characterize nanoparticles, particularly extracellular vesicles (EVs). FluoMDS enables EV detection, sizing, separation, and biomarker analysis by combining nonspecific staining of lipids and proteins with targeted fluorescent antibody labeling of EV markers. This method can determine the average size of EV subpopulations and quantify free *versus* bound antibodies in a single-step immunoassay.

## 4. Technical developments

Since its inception, MDS has evolved through a series of key technological innovations that have broadened its scope and applicability. This section highlights major advances that enhance its sensitivity, versatility, and integration into multidimensional biophysical analyses.

### 4.1. Label-free analysis

**Fig. 3:**
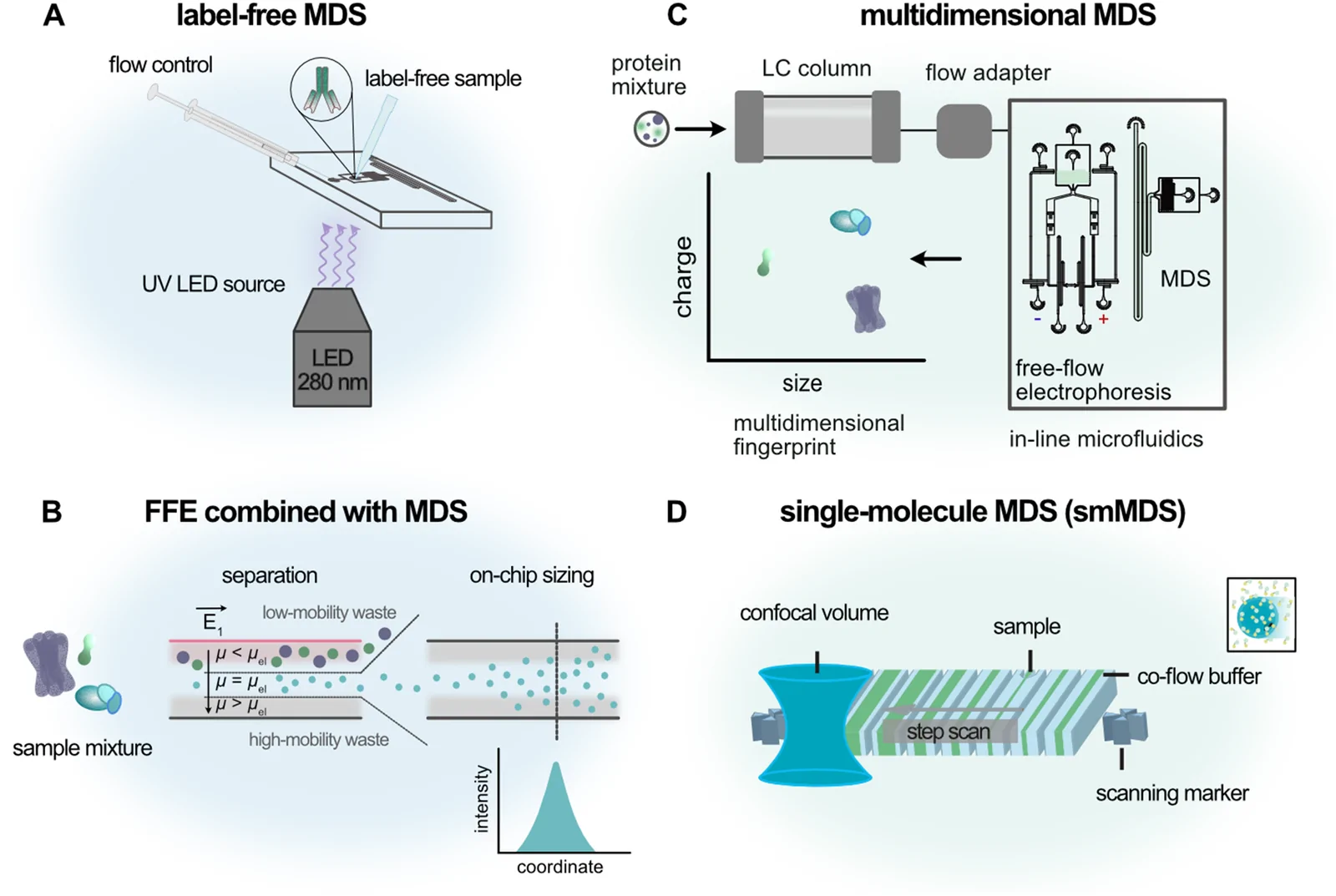
Technical advances of MDS. (A) Label-free MDS.^132^ This setup exploits the intrinsic UV fluorescence of proteins, which arises from aromatic residues such as tryptophan and tyrosine, to avoid labeling and purification steps. A UV light-emitting diode (LED) providing excitation at 280 nm was integrated into an MDS device, enabling label-free analysis. Adapted from ref. 132 with permission from the American Chemical Society (ACS). Copyright © 2018, ACS. (B) Free-flow electrophoresis (FFE) with MDS.^136^ In this setup, analysis with MDS is expanded to include charge information. FFE was integrated into the MDS platform, enabling simultaneous quantification of size and charge in heterogeneous mixtures. Before particles are characterized by MDS, FFE fractionates analytes based on their electrophoretic mobility. By varying the applied voltage, molecules are separated and directed to the MDS sizing unit on the same chip. Adapted from ref. 136 under a CC BY 4.0 license. Copyright © 2019, the authors. (C) Multidimensional MDS.^137^ As a further development, this approach was extended by combining MDS and FFE with liquid chromatography. The protein mixture is first separated by LC before passing through the FFE and MDS units on a single chip, resulting in a multidimensional fingerprint. With this configuration, the hydrodynamic radius, electrophoretic mobility, effective molecular charge, and isoelectric point of protein species can be determined. Adapted from ref. 137 under a CC BY-NC 3.0 license. Copyright © 2020, the authors. (D) Single-molecule MDS (smMDS).^6^ This setup integrates a confocal fluorescence detection scheme into the MDS platform, enabling analysis down to the single-molecule level. At each channel position, individual analyte molecules are detected and counted, generating diffusion profiles that are analyzed with an advection–diffusion model, as in conventional MDS, to determine hydrodynamic sizes. Owing to its digital fluorescence detection and single-molecule sensitivity, smMDS is five orders of magnitude more sensitive than conventional MDS and can detect biomolecules down to the femtomolar range. It also resolves the size distribution of heterogeneous samples, such as protein oligomers. Adapted from ref. 6 under a CC BY 4.0 license. Copyright © 2024, the authors.

Conventional implementations of MDS typically require labeling the analyte with extrinsic fluorophores to enhance signal detection. However, this labeling process is time-consuming, often requires purification steps, and can perturb the native behavior of the system due to interactions between the fluorophore and the analyte. To overcome these limitations, a label-free MDS platform^132^ was developed that exploits the intrinsic ultraviolet (UV) fluorescence of proteins, primarily arising from aromatic residues such as tryptophan and tyrosine (Fig. 3A). The authors integrated 280 nm excitation using a UV light-emitting diode (LED) into a microfluidic MDS device, enabling direct visualization of protein diffusion without external labeling. This system allowed MDS of proteins such as BSA, lysozyme, and native αB-crystallin complexes in solution. The approach achieves sensitivity in the high nanomolar range, providing an alternative to conventional fluorescent labeling methods while preserving native conditions.

### 4.2. Alternative signal readout strategies

While fluorescence-based detection remains the primary and most widely used readout strategy for MDS, alternative signal modalities have been explored in related diffusion-based microfluidic systems and may be adaptable to MDS applications. Brightfield and phase-contrast microscopy can visualize concentration gradients of non-fluorescent analytes in co-flow devices, although sensitivity is generally limited to millimolar concentrations, restricting their utility for biomolecular analysis.^133^ Refractive-index-based detection, including integrated photonic crystal sensors embedded in H-filter geometries, has enabled label-free monitoring of diffusion profiles for non-absorbing analytes such as glucose and BSA.^134^ Absorbance-based detection at 280 nm, which is closely related to the UV-fluorescence approach described above,^132^ can be used to monitor protein diffusion but suffers from limited optical path lengths and low sensitivity in shallow microfluidic channels. Electrical detection methods, including impedance spectroscopy and capacitively coupled contactless conductivity detection (C^4^D), have been applied in microfluidic separation systems but have not yet been translated to diffusional sizing applications.^135^ Scattering-based detection, while central to techniques such as DLS, remains challenging in co-flow MDS geometries as nanometer-scale particles exhibit weak scattering signals over the short optical path lengths characteristic of microfluidic devices. Overall, non-fluorescent detection strategies for MDS remain largely underexplored but represent an important direction for extending diffusional sizing to unlabeled analytes and facilitating implementation in point-of-care settings where dedicated fluorescence instrumentation may not be available.

### 4.3. Multidimensional protein characterization

MDS provides unidimensional information, namely molecular size, which limits its ability to analyze complex mixtures. To expand the accessible parameter space, a microfluidic platform for multidimensional analysis was developed by integrating MDS with microfluidic free-flow electrophoresis (FFE).^136^ This combination enables the simultaneous quantification of size and charge in heterogeneous mixtures. First, on-chip FFE is performed to fractionate analytes based on their electrophoretic mobility (Fig. 3B). By varying the applied voltage, molecules with defined mobilities are directed to the downstream diffusional sizing unit on the same chip. The authors used this platform to resolve a protein mixture (BSA, lysozyme) containing components with similar sizes that could not be distinguished using sizing alone. Furthermore, they demonstrated that the platform enables rapid two-dimensional fingerprinting of these heterogeneous protein mixtures in tens of seconds, paving the way for multiparameter analysis of biomolecular systems on a minute timescale.

In a further development,^137^ this approach was extended by combining MDS and FFE with liquid chromatography for multidimensional characterization of biomolecules in complex solution-phase mixtures (Fig. 3C). Following chromatographic separation, a small fraction of the flow-through is simultaneously directed to both MDS and FFE units. This configuration allows the determination of hydrodynamic radius, electrophoretic mobility, effective molecular charge, and isoelectric point of isolated protein species. Using this integrated setup, the authors successfully resolved a mixture of streptavidin interacting with biotinylated BSA and fluorophores, forming stable complexes with diverse stoichiometries and biophysical properties. This platform could provide a tool for characterizing multidimensional physical properties of proteins in heterogeneous mixtures, including complex biological samples such as serum or lysates, aggregation-prone mixtures, and chaperone–client systems.

### 4.4. Single-molecule sizing

A recent study extended the analyte concentration range accessible through MDS to the single-molecule level.^6^ Single-molecule MDS (smMDS) enables measurement of the hydrodynamic radius by detecting individual protein molecules, enabling ultrasensitive sizing down to femtomolar concentrations (Fig. 3D). smMDS further allows for the determination of binding affinities from a series of single-molecule measurements, as demonstrated for antigen–antibody interactions. This ability is particularly valuable for affinity profiling of antibody interactions at ultralow concentrations. An advantageous feature of smMDS is its ability to resolve the size distributions of heterogeneous protein assemblies, such as α-synuclein oligomers. Moreover, smMDS can distinguish multiple protein species within complex aggregation mixtures. For example, the authors used the method to characterize the sizes of fibrillar protein aggregates and nanoscale condensate clusters within a background of high monomer concentration, demonstrating that smMDS can provide size information on coexisting assembly states within heterogeneous systems, even when one species is present in large excess. Overall, smMDS offers broad applicability in biological and biomedical research, with strong potential for analyzing protein interactions in drug discovery, diagnostics, and nanobiotechnology.

## 5. Conclusion and future perspectives

MDS has evolved into a versatile tool in modern bioanalysis and biosensing, providing quantitative insights into molecular size and interactions in solution. Its unique ability to operate under native conditions, with minimal sample consumption and in complex biological matrices, distinguishes it from traditional biophysical methods. Over the past decade, MDS has demonstrated its broad applicability across a wide range of molecular systems—from protein–protein and protein–nucleic acid interactions, to lipid–protein assemblies, and to antibody affinity profiling in crude samples. The growing body of applications has not only expanded the methodological portfolio of MDS but has also showcased its capacity to answer biologically and clinically relevant questions.

With recent technical advances, including universal labeling, label-free intrinsic fluorescence detection, and multidimensional integration with electrophoresis and chromatography, MDS is poised to enter a new phase of capability and precision. These innovations dramatically enhance the method's resolution and throughput, making it suitable for the characterization of polydisperse, heterogeneous, and aggregation-prone systems. The development of universal fluorescent labeling strategies, including the use of environment-sensitive dyes, has also lowered the barriers to adoption in complex diagnostic and therapeutic contexts. Furthermore, smMDS extends the power of this approach to ultralow concentrations and provides access to the detailed size distribution of coexisting species.

Despite its advantages, MDS faces several challenges. Although heterogeneous size distributions can now be resolved using smMDS, they remain difficult to characterize with conventional MDS. Moreover, the accessible size range depends on the device geometry: H-type MDS typically covers approximately 1–25 nm, whereas T-type MDS can extend to approximately 1–250 nm. These ranges are well suited to PPIs and many other biomolecular interactions; however, further methodological development to broaden the accessible size range would enable more reliable analysis of both smaller ligands and larger assemblies such as vesicles. Furthermore, the use of fluorophores can pose issues if they disrupt the interaction under study. In such cases, it is advisable to label the analyte only after separating the sample and buffer streams.

Practical considerations surrounding cost, accessibility, and standardization will also influence the broader adoption of MDS technologies. Commercial MDS instruments, such as the Fluidity One-M (Fluidic Sciences), represent a moderate capital investment compared to high-end biophysical equipment such as AUC, SEC-MALS, or SPR systems. However, the cost of disposable microfluidic chips adds a recurring per-sample cost that can become significant in high-throughput screening applications. By contrast, DLS and MST instruments, while often comparable in initial investment, do not require consumables. Custom-built T-type MDS setups offer greater flexibility and lower consumable costs but require microfluidic fabrication expertise and bespoke optical detection setups, limiting their accessibility to specialized laboratories.

The requirement for fluorescent labeling of at least one binding partner further represents a practical barrier for certain analytes and applications. Moreover, broader implementation of MDS in clinical and pharmaceutical settings will require improved standardization of microfluidic devices, measurement protocols, and data analysis workflows across laboratories. Future development of integrated and automated platforms with user-friendly analysis software is therefore expected to play an important role in expanding the accessibility and translation potential of MDS. Clinical translation will additionally require validated and standardized measurement protocols, inter-laboratory reproducibility studies, and formal comparison with established diagnostic assays under regulatory frameworks.

Looking ahead, the integration of MDS with automated sample handling and miniaturized detection optics could further streamline its application in both research and clinical laboratories. Machine-learning approaches may prove particularly useful for deconvolving multimodal size distributions from diffusion profiles, for automated quality control of raw fluorescence data, and for training surrogate models that accelerate the numerical solution of the advection–diffusion equation during fitting. Additionally, translation of diffusion-based sizing principles to low-cost substrates such as paper-based microfluidic networks,^138^ including implementations inspired by T-sensor and H-filter concepts, could substantially reduce per-sample costs and facilitate deployment in resource-limited settings.

We anticipate that MDS will play an increasingly central role in precision diagnostics, therapeutic development, and personalized medicine, where the ability to quantify biomolecular interactions directly in complex fluids such as serum or plasma is critical. Its compatibility with native-state analyses, minimal sample perturbation, and quantitative rigor make MDS well suited to address emerging challenges in molecular biosciences, especially at the interface of synthetic biology, nanomedicine, and immunotherapy.

## Conflicts of interest

There are no conflicts to declare.

## Data Availability

No primary research results, software or code have been included and no new data were generated or analyzed as part of this review.
